# A Comprehensive Mechanical Testing of Polyacrylamide Hydrogels: The Impact of Crosslink Density

**DOI:** 10.3390/polym17060737

**Published:** 2025-03-11

**Authors:** Christina G. Antipova, Arthur E. Krupnin, Arthur R. Zakirov, Vsevolod V. Pobezhimov, Daniil A. Romanenko, Dina Yu. Stolyarova, Sergei N. Chvalun, Timofei E. Grigoriev

**Affiliations:** 1 NRC Kurchatov Institute, pl. Akademika Kurchatova 1, 123098 Moscow, Russia; krupnin_ae@nrcki.ru (A.E.K.); pobezhimov_vv@nrcki.ru (V.V.P.); zver.romanencko2012@yandex.ru (D.A.R.); stolyarova_dy@nrcki.ru (D.Y.S.); chvalun_sn@nrcki.ru (S.N.C.); grigoriev@nrcki.ru (T.E.G.); 2 MIREA—Russian Technological University, Vernadsky Avenue 86, 119571 Moscow, Russia; bruddddder@gmail.com; 3 Enikolopov Institute of Synthetic Polymeric Materials, Profsoyuznaya 70, 117393 Moscow, Russia

**Keywords:** polyacrylamide hydrogels, hydrogel mechanical behavior, indentation, hydrogel torsion, tension, compression, FEA

## Abstract

Mechanical properties are one of the most important characteristics of biomaterials for many different applications, including biomedicine. Soft biomaterials, such as hydrogels, are difficult to characterize by conventional mechanical testing, because their mechanical properties are much lower than required by conventional testing machines. In this work, we aimed to systematically study the mechanical behavior of a model soft material, polyacrylamide hydrogels, under different loading modes: tension, torsion, compression, and indentation. This allowed us to develop a comprehensive approach to the mechanical testing of soft materials. To overcome excessive compression and slippage of the hydrogel samples when fixed in the grips during tension, additional 3D-printed grips were designed. Digital image correlation was used to determine the Poisson’s ratio of the hydrogels. The Young’s modulus values obtained from all types of mechanical tests analyzed were highly correlated. However, for hydrogels with a low crosslinker concentration, 1–2%, tension–compression asymmetry was observed. Moreover, the results of the mechanical tests were verified in indentation tests, including analytical estimation, and full-scale and numerical experiments. We also discuss the limits of using a two-parameter Mooney–Rivlin model for fitting hydrogel uniaxial tension deformation curves, which was unstable for the hydrogels with 4 and 9% crosslinker concentration. The implemented approach provided a comprehensive analysis of the mechanical behavior of biomaterials. The elastic moduli for all hydrogels studied were in the range from 20 to 160 kPa, which corresponds well to human soft tissues, making them a promising material for application as tissue-mimicking phantoms.

## 1. Introduction

Hydrogels are three-dimensional polymer networks capable of retaining large amounts of water due to hydrophilic functional groups in their structure. Hydrogels are well known in various biomedical applications because they exhibit biocompatibility and closely mimic the native extracellular matrix [1]. For example, natural and synthetic polymer-based hydrogels are used as carriers in drug delivery [2], scaffolds mimicking soft tissues in tissue engineering [3], wound healing dressings [4], tissue phantoms [5], and coating layers for implantable medical devices [6]. In all these applications, the mechanical properties of the hydrogel play an important role, as they define the interaction of the implantable device with the surrounding tissues; provide adequate functioning of the healing area during regeneration; and, equally important, affect cell motility, adhesion, proliferation, and differentiation [7].

The Young’s modulus of hydrogels is typically in the range of 1–200 kPa, which on the one hand resembles native soft tissues, but on the other hand causes difficulties in mechanical testing. Because hydrogels are much softer than common materials such as ceramics, metals, and plastics, not every conventional test may be suitable for determining their mechanical behavior [8]. For example, hydrogels slip, break easily, and deform under their own weight, so measuring the mechanical properties of hydrogels using bending and tensile tests is challenging [9]. To overcome this problem, various fixation techniques, including additional grips [10,11], have been proposed.

Another peculiarity of the mechanical properties of hydrogels is their nonlinear behavior over a large strain range [12]. To describe such behavior, one can use numerous constitutive hyperelastic models, which assume the time-independency and full-reversibility of the strains being applied after unloading [10]. Among them, neo-Hookean and 2-parameter Mooney–Rivlin models are the most simple and, at the same time, have a clear physical interpretation of coefficients, corresponding to the initial shear modulus G [13].

Since hydrogels may be too soft for conventional mechanical testing machines, it is important to use a cross-evaluation approach to characterize the mechanical behavior of hydrogels, and to validate the results of one testing method by another. Despite a large number of papers investigating the mechanical properties of hydrogels, the vast majority used a specific method for each characterization parameter (e.g., stiffness, Young’s modulus, ultimate tensile stress, or strain, etc.). Only a limited number of articles have addressed this issue and provided results for the same mechanical parameter determined using different testing methods [9,12,14,15]. Stammen [12] used two alternative approaches to evaluate the compressive failure of polyvinyl alcohol hydrogels. The papers by Gandin [9] and Buffinton [15] focused on the validation of alternative techniques for measuring the elastic moduli of hydrogels, namely static macroindentation in the first case, nanoindentation in the second, and micropipette aspiration in both articles. Validation was carried out by comparing the results obtained from different mechanical tests (i.e., tension, compression, atomic force microscopy, and rheology). To the best of our knowledge, the works of Czerner [8] and Richbourg [14] were the first to provide a balanced comparison of a range of approaches to measuring hydrogel stiffness, using as model gelatin- or polyvinyl-alcohol-based hydrogels.

In our work, we aimed to continue exploring the mechanical properties of hydrogels under different types of loading, extend the scope of knowledge in the field, and propose several useful adjustments to mechanical testing and validation processes.

To that end, we wanted to pay more attention to indentation. Indentation techniques have effectively been used to establish the mechanical properties of biomaterials using elastic, viscoelastic, or poroviscoelastic theories. This is due to their simplicity, as well as the inability to use alternative experimental methods for in vivo or in vitro mechanical testing. Thus, Xu [16] utilized a poroelastic model and depth profiling nanoindentation to ascertain the hydraulic conductivity and elastic moduli of thin polyacrylamide (PAAm) hydrogel layers (thickness ranging 27–782 μm) on impenetrable substrates. The apparent stiffness of slim PAAm layers rose with indentation depth and was considerably higher compared to their thicker counterparts, which showed no impact of indentation depth. Reinhards [17] determined the impact of crosslinker concentration on the mechanical properties of polyacrylamide-based hydrogels via depth-sensing indentation. Viscoelasticity and poroelasticity at intermediate length scales were taken into account in the study by considering hydrogels as poroviscoelastic solids. A constrained fitting technique was formulated to implement a multiplicative rule that factored in the influence of every deformation mechanism on the overall material response. The proposed technique was resilient enough to accurately differentiate poroelastic and viscoelastic contributions from the relaxation curves obtained at various indentation depths and strain rates. At the length scales tested, viscoelasticity dominated, while the poroelastic contribution became more significant as the concentration of crosslinkers diminished.

At present, for determining the mechanical properties of soft tissues and hydrogels, a combination of full-scale indentation experiments and finite element analysis (FEA) is widely used. Kalyanam [18] assessed the mechanical properties of gelatin hydrogels via indentation-load relaxation and quasistatic indentation tests, and converted the properties obtained from a rheometer–stress relaxation experiment into a biphasic poroviscoelastic (BPVE) material model to simulate indentation tests using finite element analysis (FEA). Parametric studies utilizing FEA of an indentation–load relaxation test accurately estimated hydraulic permeability and the largest time constant. The force response predicted from FEA of indentation experiments utilizing BPE and BPVE models illustrated the significant role played by the flow-independent solid matrix relaxation behavior of soft polymeric media, emphasizing the necessity of applying a BPVE model. In studies on indentation testing of gelatin hydrogels, it has been determined that the shear relaxation modulus is an inherent material property of both soft tissues and hydrogels. This property is considered by researchers to be the most suitable for comparison with results obtained from other experiments. Haddad [19] determined the hyperelastic mechanical properties of oropharyngeal soft tissues, which included palatine tonsil, soft palate, uvula, and tongue base, and performed indentation testing on these specimens to obtain their force–displacement data. An inverse FE framework was used to establish the hyperelastic parameters of the specimens from these data. The hyperelastic parameters of Yeoh and second-order Ogden models were acquired in the study. The experimental force–displacement data of the tissue specimens were captured reasonably accurately by both models, with mean errors of 11.65% or less. The study estimated the hyperelastic parameters of all upper airway soft tissues utilizing freshly acquired human tissue specimens for the first time.

In our work, PAAm hydrogels were chosen as model materials, since they can be routinely synthesized from its monomer—acrylamide—by free-radical polymerization, and their mechanical properties can be easily modified over a wide range by varying the total polymer concentration or the crosslinker content [20]. According to literature data [1], the elastic modulus of PAAm hydrogels is in the range of 0.01 kPa–1 MPa, which significantly covers the values of soft tissues [21]. Thus, PAAm hydrogels can serve as soft-tissue-miming phantoms, materials that closely simulate tissue’s physical properties. Phantoms are widely used in the development, testing, and calibration of medical imaging techniques, advanced diagnostic and therapeutic methods, as well as being useful in planning and rehearsing surgeries, and for rehabilitation and doctor–patient communication [5,22].

In this paper, we intended (1) to systematically investigate the mechanical behavior of PAAm-based hydrogels in tension, compression, torsion, and indentation; (2) to develop a comprehensive approach for mechanical testing of soft materials, including validation and cross-evaluation of results; (3) to discuss the characteristics of full-scale and numerical experiments, as well as the applicability of analytical estimation and constitutive modeling; (4) to observe the effect of crosslinks on the mechanical response of hydrogels; and (5) to confirm that PAAm hydrogels can mimic soft tissue mechanics and, as a result, be used as phantoms. The novelty of our work can be summarized as the proposed additional grips for tensile testing; the cross-evaluation of the mechanical behavior of PAAm hydrogels, including a wide range of methods; and a deeper analysis of the mechanical phenomena appearing during mechanical experiments.

## 2. Materials and Methods

### 2.1. Synthesis

We dissolved acrylamide (2 g, Sigma-Aldrich, Burlington, MA, USA) in bidistilled water (10 mL) obtained on a Millipore Milli-Q, to obtain a monomer concentration of 20%. Various amounts of N,N’-methylenebisacrylamide (BIS) were added to the acrylamide. Crosslinker concentrations (%C=) were calculated using the following formula: $\%C=\frac{BIS[g]}{acrylamide[g]+BIS[g]}$. The selected concentrations of BIS and their ratios to the number of monomers in the precursor mixture are given in Table 1. N,N,N’,N’-tetramethyl ethylene diamine (65 μL) and ammonium persulfate (50 mg) were used as the activator and initiator, respectively, to initiate the radical polymerization. The polymerization took 15 min. After polymerization, all hydrogels were kept in distilled water for 2 weeks to achieve equilibrium swelling and to remove unreacted components. For the reaction, the conversion was more than 99%.

For the different mechanical tests, the samples were synthesized in different shapes (Table 2).

### 2.2. Mechanical Properties

#### 2.2.1. Swelling

The equilibrium swelling ratio was estimated gravimetrically for all hydrogels. Fully swollen hydrogels were weighed, then dried in a vacuum oven at 60 °C to constant mass and reweighed. The swelling ratio was calculated using the following formula [23]:

(1) $$\alpha =\frac{M_{s}-M_{d}}{M_{d}}*100\%,$$

where $M_{s}$ is the mass in the swollen state and $M_{d}$ is the mass in the dried state.

#### 2.2.2. Average Molecular Weight Between Crosslinks

Average molecular weight between crosslinks ($M_{c}$) was estimated using three different approaches. The first was based on the Flory–Rehner equation [24]:

(2) $$\frac{1}{M_{c}}=\frac{2}{M_{n}}-\frac{\log1-\phi_{s}+\phi_{s}+\chi \phi_{s}}{V_{1}\phi_{d}\left[\phi_{2}^{\frac{1}{3}}-\frac{\phi_{s}}{2}\right]},$$

where $M_{n}$ is the average number molecular mass of the polymer, $\phi_{s}$ is the polymer volume fraction at swelling equilibrium, χ is the polymer–solvent interaction parameter, $V_{1}$ is the molar volume of the solvent, and $\rho_{d}$ is the dry density of the polymer network [24].

The second equation joins the average molecular weight between crosslinks with the mechanical properties of the network [25]:

(3) $$\frac{\sigma }{\left(\lambda -1/\lambda^{2}\right)}=RT\frac{C_{2,r}}{M_{c}}\left(1-\frac{2M_{c}}{M_{n}}\right)Q^{1/3},$$

where σ is the tensile stress, λ is the extension ratio, $C_{2,r}$ is the polymer mass concentration, and *Q* is the volume swelling ratio [25].

For validation, theoretical estimation was used:

(4) $$M_{c}=N_{c}\times 71\;g\;{\mathrm{mol}}^{-1},$$

where $N_{c}$ is the number of the monomers between crosslinks based on the BIS to acrylamide ratio, and 71 g mol^−1^ is the molecular weight of BIS.

#### 2.2.3. Uniaxial Tension

**Figure 1:**
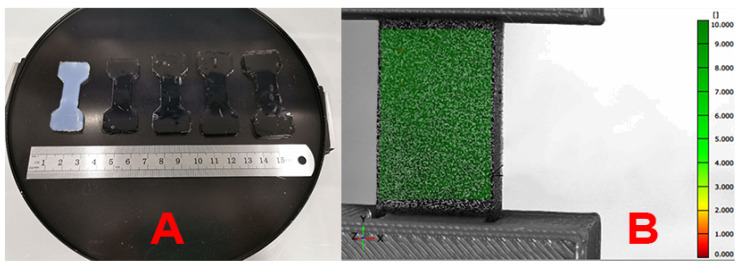
Images of the samples after swelling (**A**) and with speckles during Poisson’s ratio determination (**B**).

**Figure 2:**
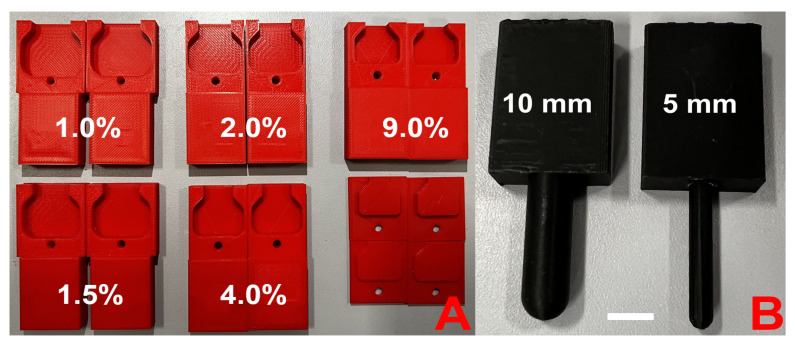
The 3D-printed (**A**) grips for uniaxial tension, (**B**) indenters with 5 mm and 10 mm diameter. The scale bar is 2 cm.

Dumbbell-shaped specimens were tested for uniaxial tension on an universal testing machine, Instron 5965 (Illinois Tool Works Inc., Glenview, IL, USA), equipped with a ±50 N load cell at a constant rate of 1 mm min^−1^ at room temperature. The number of specimens tested for each series varied from 5 to 10, to ensure maximum reproducibility of the results. To eliminate overpressure and slippage, specially designed grips were fabricated from ABS plastic using a Zortrax M200 3D printer (Zortrax, Olsztyn Poland). As the volume change of the sample during swelling was strongly dependent on the crosslinker concentration (%C) (Figure 1A), 5 different grips were designed for each hydrogel type (Figure 2A).

#### 2.2.4. Uniaxial Compression

Uniaxial compression tests of cylindrical specimens were performed on an universal testing machine, Instron 5965 (Illinois Tool Works Inc., Glenview, IL, USA), equipped with a ±50 N load cell at a constant rate of 1 mm min^−1^ at room temperature. The number of specimens tested for each series ranged from 5 to 10, to ensure maximum reproducibility of the results.

#### 2.2.5. Torsion

Torsion tests on cylindrical specimens were performed on an Anton Paar Physica MCR 501 rotary rheometer (Anton Paar GmbH, Graz, Austria) with a 50 mm parallel-plate geometry at a constant angular velocity of 1 rad s^−1^ at room temperature. To prevent the samples from slipping, sandpaper was glued to the rheometer plates. As the magnitude of the applied force affected the results, the influence of the normal force (NF) on the magnitude of the maximum shear stresses was also evaluated and the NF value was varied in the range from 1 to 4 N. The tests were carried out up to shear strain values of 10% or before the sample started to slip. The number of specimens tested for each series was between 5 and 10, to ensure maximum reproducibility of the results. From the test results, the shear modulus G was determined as follows [26]:

(5) $$G=\frac{M_{K}}{W_{p}},$$

where $M_{K}$—the magnitude of the torque on the linear sections of the torsion diagram, and $W_{p}=\frac{\pi *D^{3}}{16}$—polar resistance moment for a sample with a diameter *D*.

After that, the Young’s modulus was determined from the known relation for an isotropic material [27]:

(6) E=2G(1+ν).

#### 2.2.6. Poisson’s Ratio

In order to evaluate the incompressibility of the obtained hydrogels and to determine the relation between the elastic modulus and the shear modulus, the Poisson ratio ν was evaluated in uniaxial tensile tests using the digital image correlation method [28]. A grid (black matte enamel paint) was stochastically applied to the surface of the gauge length of a dumbbell specimen and a set of images were obtained with a resolution (4032 × 3024) in the linear region of the stress–strain dependence, with a constant time step of 0.5 s. The transverse ($\epsilon_{x}$) and longitudinal ($\epsilon_{y}$) strain fields were plotted using GOM Correlate software (Zeiss Group, Oberkochen, Germany). During image processing, the coordinate axes were oriented to avoid distortion of the results due to a non-perpendicular camera installation with respect to the sample surface. Poisson’s ratio was defined as the absolute value of the ratio of transverse strain $\epsilon_{x}$ to longitudinal strain $\epsilon_{y}$ [29]:

(7) $$\nu =|\frac{\epsilon_{x}}{\epsilon_{y}}|.$$

#### 2.2.7. Indentation

Spherical indenters are widely used to determine the local mechanical properties of soft biological tissues, as the absence of sharp edges reduces the risk of tissue damage [30]. Therefore, cylindrical specimens with a nominal (before swelling) diameter of 50 mm and a height of 30 mm were indented using two spherical indenters of 5 and 10 mm diameter, fabricated on a Zortrax M200 3D printer (Zortrax, Olsztyn, Poland) from ABS plastic (Figure 2B). The tests were carried out on a universal testing machine, Instron 5965 (Illinois Tool Works Inc., Glenview, IL, USA), equipped with a ±50 N load cell at a constant speed of 1 mm min^−1^ at room temperature. The indentation depth was chosen to be equal to the radius of the indenter used in the test. The overall dimensions of the specimens were chosen to minimize the influence of thickness and edge effects on the experimental results in the theoretical evaluation of the elasticity parameters obtained in the uniaxial tension and torsion experiments.

Analytical prediction was carried out using [31,32]:

(8) $$P=\frac{8\sqrt{R\delta^{3}}G}{3(1-\nu )},$$

where *P* is the reaction force, *R* is the indenter’s radius, δ is the indentation depth, *G* is the shear modulus, and ν is the Poisson’s ratio of the hydrogel.

The accuracy of the experimental data was estimated by the following formula [33]:

(9) $$e=\frac{1}{N}\Sigma \left|\frac{y_{exp}-y}{y_{exp}}\right|,$$

where *N*—the number of experimental points, $y_{exp}$—the experimental value of the force, and *y*—the analytically predicted value of the force.

#### 2.2.8. Hyperelastic Models

The nonlinear deformation behavior of hydrogels can be described by hyperelastic models [10]. Currently, the most commonly used models are neo-Hookean, Mooney–Rivlin, Ogden, Gent, Yeoh, etc. [34]. However, higher-order models require the determination of constants that do not have an explicit and clear physical meaning. The neo-Hookean and two-parameter Mooney–Rivlin models have no such drawbacks, where the constants are explicitly related to the shear modulus G, which led to their selection in this paper. These models were used to fit stress–strain data from uniaxial tensile tests. For all models, the shear modulus was followed during fitting, comparing the results obtained by the different models and in tension and torsion.

##### Neo-Hookean

The neo-Hookean (NH) model is the simplest material model describing hyperelastic behavior. For an incompressible material, the NH strain energy density is equal to the following expression [13]:

(10) $$W=C_{1}\left(I_{1}-3\right),$$

where $C_{1}$ is a material constant and $I_{1}$ is the first invariant of the left Cauchy–Green deformation tensor.

The shear modulus explicitly enters the Cauchy stress expression for uniaxial deformation [13]:

(11) $$\sigma =G(\lambda -1/\lambda^{2}),$$

where *G* is the shear modulus, and λ is the principle stretch ratio.

The neo-Hookean model adequately represents the initial linear region of a stress–strain curve for rubber-like materials, including crosslinked polymers and hydrogels. However, it does not predict their mechanical behavior at moderate and large strains (more than 50%) [13].

##### Mooney–Rivlin

The 2-parameter Mooney–Rivlin (MR) model is another conventional expression for predicting the stress–strain behavior of materials, now based on two strain invariants. The strain energy density includes their linear combination and, in the case of an incompressible material, has the following form [35]:

(12) $$W=C_{10}\left(I_{1}-3\right)+C_{01}\left(I_{2}-3\right),$$

where $C_{10}$ and $C_{01}$ are material constants, and $I_{1}$ and $I_{2}$ are the first and the second invariants of the left Cauchy–Green deformation tensor, respectively. Unlike the neo-Hookean equation, the shear modulus is expressed as $G=2(C_{10}+C_{01})$.

In the case of uniaxial tension, the Cauchy stress is expressed as [35]

(13) $$\sigma =2\left(\lambda -1/\lambda^{2}\right)\left(C_{10}+\frac{C_{01}}{\lambda }\right).$$

The MR model can improve the prediction of the NH model at moderate and large strains, and is broadly used for rubber-like materials, including crosslinked polymers and hydrogels [35].

#### 2.2.9. Finite Element Analysis

**Figure 3:**
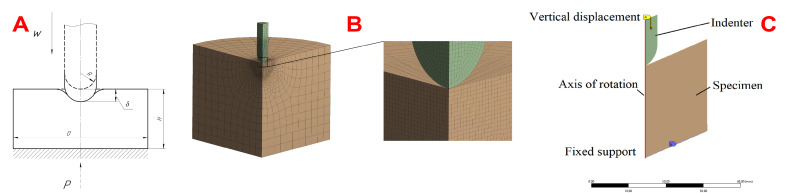
Numerical indentation experiment: (**A**) plane model with geometric parameters, (**B**) finite element mesh for the 90 degree sector, (**C**) boundary conditions.

In order to verify the elasticity parameters of the hydrogels determined from the tensile and torsion tests, a numerical simulation of the indentation was carried out using the finite element method (FEM) in the ANSYS Workbench software package 2021 R1 (Ansys Inc., Canonsburg, PA, USA). The material was assumed to be isotropic when solving the axisymmetric indentation problem. Neo-Hookean, two-parameter Mooney–Rivlin models were used to describe the hyperelastic behavior of the hydrogels. The constants obtained from the results of fitting the uniaxial tension curves were used for the numerical experiment. The indenter material was assumed to be absolutely rigid. During the model design, the diameter *D* and the height *H* of cylindrical specimens were measured to evaluate the effect of geometry changes after swelling. The following boundary conditions were used: the lower surface of the specimen was fixed in all degrees of freedom, and a vertical displacement equal to the radius of the indenter used was applied to the upper face of the indenter (Figure 3A). Based on the results of the mesh convergence test, the number of 8-node PLANE183 elements and nodes varied from 7487 to 8305 and from 23,030 to 25,510, respectively, depending on the geometric dimensions of the specimens and the indenter (Figure 3B). The element size in the refinement area of the mesh was 0.2 mm (Figure 3C). The augmented Lagrange method was used to simulate frictionless contact between the indenter and the specimen. The numerical force–displacement curves were compared with the experimental curves.

### 2.3. Biological-Tissue-Mimicking Phantom Manufacturing

Spleen, kidney, and liver models were taken from turbosquid.com. Their processing (general remeshing and smoothing) was carried out in Autodesk Meshmixer (Autodesk, San Francisco, CA, USA). Mold design was performed in Autodesk Inventor, and Boolean subtraction of the organs from the base geometry was performed in Autodesk Netfabb, taking into account the swelling ratios of the hydrogels. The 3D printing was performed on an Elegoo Neptune 3 Plus 3D printer (Elegoo Inc., Shenzhen, China) using a commercial acrylonitrile-butadiene-styrene-based filament from FDplast (Moscow, Russia). Post-processing included grinding and chemical polishing with acetone of the inner surfaces of the molds. The half molds were mounted on M3 countersunk screws.

**Figure 4:**
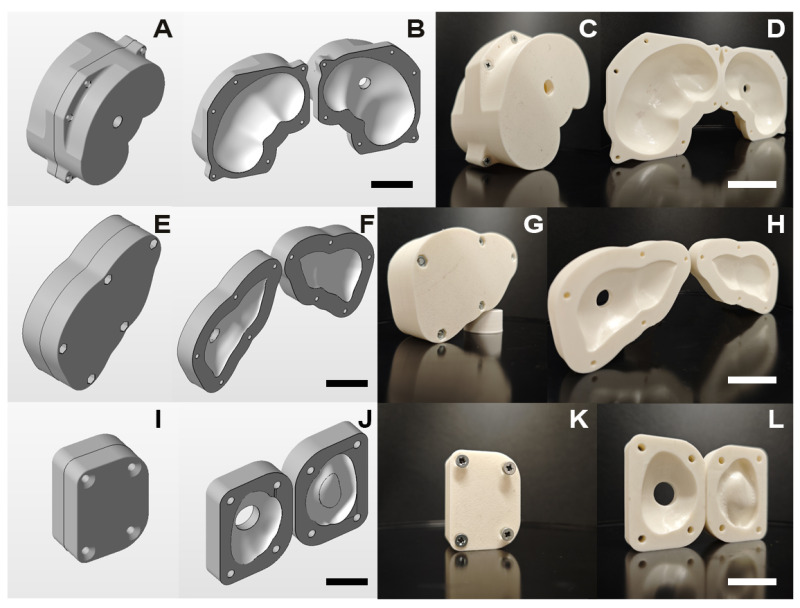
Models of phantom molds: (**A**,**B**) kidney, (**E**,**F**) liver, (**I**,**J**) spleen; manufactured phantom molds: (**C**,**D**) kidney, (**G**,**H**) liver, (**K**,**L**) spleen. The scale bar is 2 cm.

The models and 3D-printed molds are shown in Figure 4.

The phantom prototypes were prepared according to the described synthesis process. For the kidney, liver, and spleen phantoms, the BIS amount was 9.0%, 4.0%, and 1.5%, respectively. To obtain more representative images, the hydrogels were swelled in dyed water.

## 3. Results

The mechanical behavior of the hydrogels was studied in different tests, including torsion, uniaxial tension, and compression. The Poisson’s ratio was estimated in a contactless method using a series of photographs showing the tensile process. The results were verified by indentation, which consisted of three stages: an experiment on the testing machine, an analytical calculation, and a numerical experiment. Indentation was chosen for verification because it implements a complex stress state, including stretching, compression, and shear [10]. All tests were performed on the equilibrium swollen specimens. The mass swelling coefficients are given in Table 1. The swelling test results correlated with the literature-based data: the swelling degree was inversely proportional to the amount of crosslinker [36].

The average molecular weights between the crosslinks estimated according to the different approaches are presented in the Table 3.

The results are in good correlation with each other. In contrast to the other samples, for the hydrogels with 9% crosslinking, the estimation based on swelling gave a result closer to the theoretical one, which may have caused by a restricted viscoelastic flow [37].

### 3.1. Uniaxial Tension

**Figure 5:**
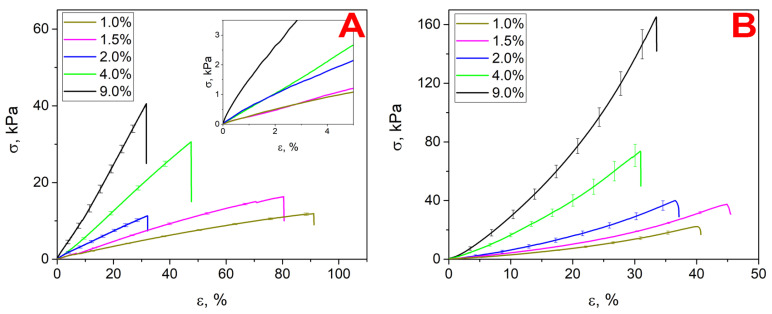
(**A**) Uniaxial tension and (**B**) uniaxial compression deformation curves.

The tensile curves of the obtained materials are shown in Figure 5A. Each of the hydrogels were characterized by an initial linear region on the curve. The Young’s modulus of the hydrogels was evaluated from the tensile curves as the slope of the deformation curve’s linear area. The Young’s modulus increased 8-fold with increasing crosslinker concentration.

The strength of the hydrogels decreased, when %C increased (Table 4). Strechability had a general tendency to increase with decreasing concentration, but remained constant in a range from 1% to 2%.

### 3.2. Uniaxial Compression

The compression curves are shown in Figure 5B. Each of the hydrogels had an initial linear section on the curve, the slope of which was used to determine the Young’s modulus. The comparison of Young’s modulus estimated from the tension and compression tests indicated a change in the symmetry of the mechanical properties at different concentrations of the crosslinker. For the highly crosslinked hydrogels (4% and 9%), a good symmetry was observed, while for lower crosslinker concentrations (1–2%), there was tension–compression asymmetry. This could have been caused by viscoplastic flow in loosely crosslinked networks, due to the presence of water molecules between the chains. In dense networks, which swell a small amount, the viscoelastic flow is restricted [37]. Similarly to the results for uniaxial tension, the strength of the hydrogels decreased when %C increased (Table 5), since the stretchability had a general tendency to increase with decreasing concentration.

### 3.3. Torsion

**Figure 6:**
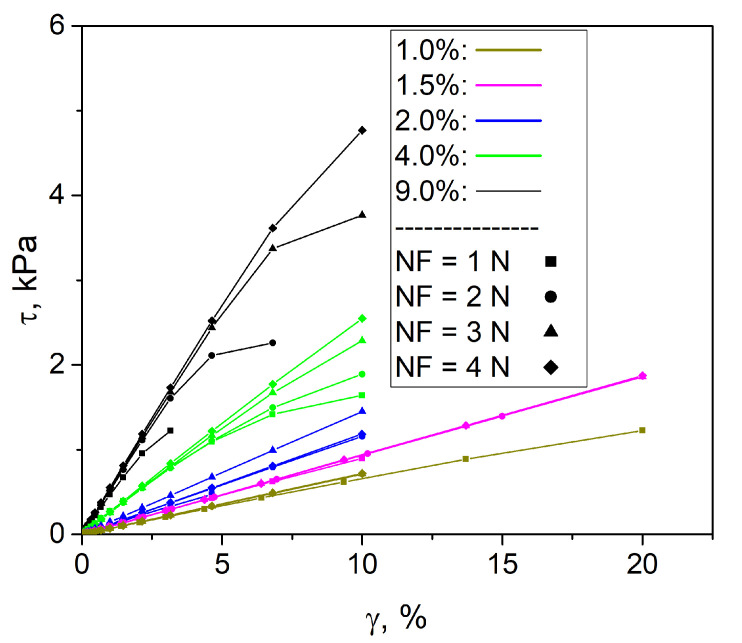
Torsion deformation curves for the hydrogels at the different initial normal force values.

According to the results of the torsion tests, it was found that increasing the value of the NF from 1 to 4 N did not lead to a change in the deformation curve for all concentrations of the crosslinker. Figure 6 shows the deformation curves at different initial forces applied to the hydrogels.

The shear modulus *G* was calculated from the deformation curves for all hydrogels. It is well known that shear modulus is related to Young’s modulus by Formula (6).

Table 5 lists the values of shear and Young’s modulus calculated taking into account the values of *G* and ν obtained in the previous experiments.

For all crosslinker concentrations, the Young’s modulus obtained in the uniaxial tensile tests coincide with the values calculated in the torsion tests.

### 3.4. Poison’s Ratio

Table 4 shows the obtained values for Poisson’s ratio. They varied between 0.477 and 0.496, indicating the incompressibility and hyperelastic behavior of the hydrogel based on polyacrylamide.

### 3.5. Indentation

**Figure 7:**
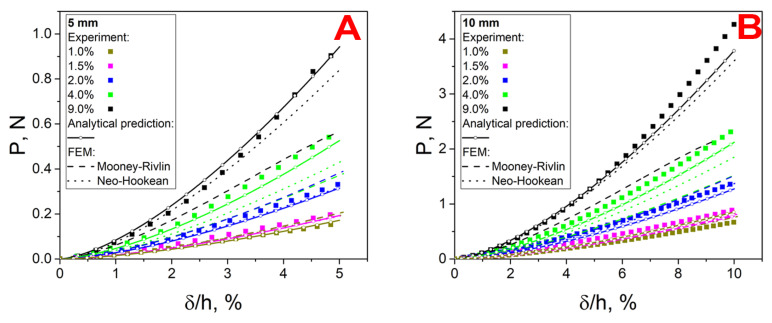
Indentation curves of the hydrogels: (**A**) indenter with d = 5 mm, (**B**) indenter with d = 10 mm.

The nonlinear dependence of the force on the indentation depth is shown in Figure 7. It can be seen that the crosslinker concentration significantly affected the mechanical response of the system, which was confirmed by the results of the tension, compression, and torsion tests. The results of the full-scale experiment were complemented by the results of the analytical calculation. It can be seen that the elasticity theory relations predicted the mechanical response of the system for different values of indenter diameters and relative indentation depths, up to 7.5% for a diameter of 5 mm and 10% for a diameter of 10 mm. The discrepancy between the analytical and experimental results in the above ranges was due to the following factors: the predominance of compressive stress over tensile stress, which required substitution of the elastic modulus determined from the results of the compression tests into the analytical ratio, and the effect of the finite thickness of the specimen at large deformations for the 10 mm indenter. For the samples with the crosslinker concentration of 1%, there was a significant discrepancy between the analytical calculation and the experimental results, regardless of the indenter diameter. In addition, stress relaxation appeared to make a significant contribution. Despite this, it can be concluded that the theoretical relationships were applicable during indentation, and the elastic modules determined from the tensile and torsion test results were correct. The maximum deviation between the calculation and experiment was 16% for the crosslinker concentration of 9%.

### 3.6. Hyperelasticity Models

**Figure 8:**
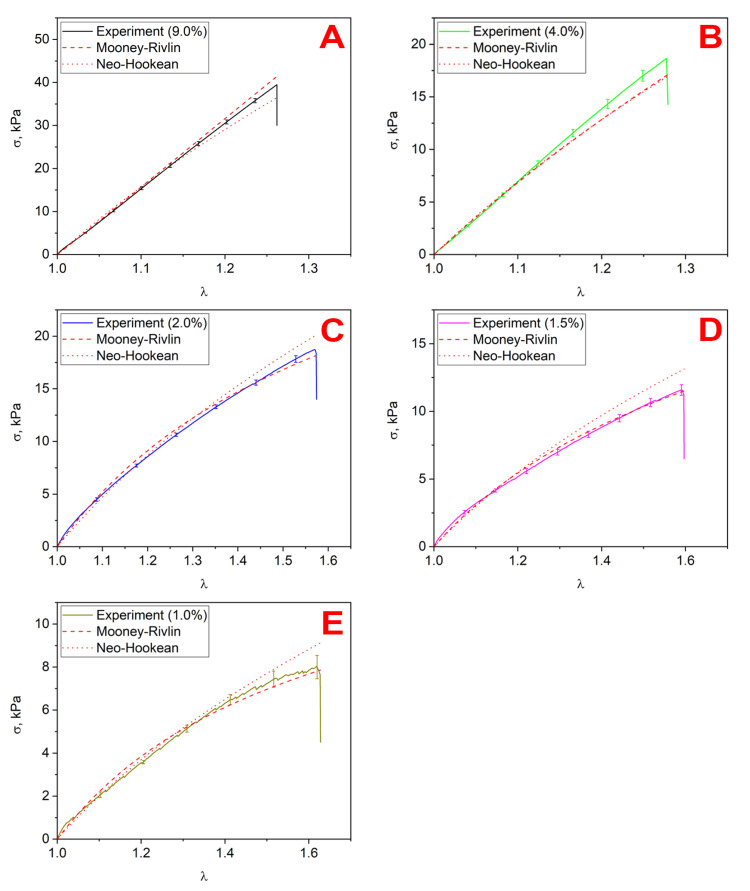
The experimental curves fitting results: (**A**) 9.0%, (**B**) 4.0%, (**C**) 2.0%, (**D**) 1.5%, (**E**) 1.0%.

The uniaxial tensile curves were fitted by the neo-Hookean and two-parameter Mooney–Rivlin hyperelasticity constitutive relations using the least squares method. The target parameter in the neo-Hookean model was the shear modulus *G*, and the coefficients $C_{10}$ and $C_{01}$, in the Mooney–Rivlin model, which also represent the shear modulus *G*, as mentioned in the expression above. The values of the fitting parameters are given in Table 6. It can be seen that, for the crosslinker concentrations of 9% and 4%, the values of the constants $C_{10}>0$ and $C_{01}<0$, while for the concentrations of 2%, 1.5%, and 1%, both constants were strictly greater than zero. This was due to the fact that the relation was used to fit the results of a single experiment (only tension) but included two unknown constants. Nevertheless, the models described the experimental curves for uniaxial tension well. Artifacts can occur when these models are applied in cases with a complex stress state, which will be examined in detail below. Figure 8 shows the original averaged uniaxial tension curves and the results of fitting with the neo-Hookean and Mooney–Rivlin models. It can be seen that the selected constants of the Mooney–Rivlin model described the hyperelastic behavior of the materials under uniaxial tension well, regardless of the sign of the $C_{01}$ constant. The constants and values of the shear modulus obtained from the approximation of the experimental curves are given in the Table 6. The data are in good agreement with the uniaxial tension and torsion results.

### 3.7. FEM

Based on the results of the numerical indentation experiment, force–indentation depth curves P=P(δ) were plotted for the neo-Hookean and Mooney–Rivlin models (Figure 7) for indenters of both diameters. The neo-Hookean model described well the mechanical response of the system at small deformations (up to 10%). As in the case of the analytical relation, the discrepancy for samples with a concentration of 1% was caused by stress relaxation in the material under quasi-static loading. For the two-parameter Mooney–Rivlin model, there was a significant discrepancy between the results of the natural and numerical experiments for concentrations of 2% and 1% in the strain range up to 2.5% for the 5 mm indenter and up to 5% for the 10 mm indenter. For the crosslinker concentration of 1.5%, a good correlation was observed between the experiment and the FEM calculation over the entire strain range. This was due to the higher value of the calculated shear modulus G (compared to the values obtained from the tensile and torsion tests). Thus, the calculated curve intersects the experimental one, thus maintaining a low value of average error. This trend continued for the curves obtained for the 2% crosslinker concentration. In the case of the hydrogel with 1% crosslinker, the Mooney–Rivlin model gave an overestimated value, which can also be justified by the contribution of stress relaxation in the natural experiment. The opposite situation was observed for materials with crosslinker concentrations of 4 and 9%. Over the entire range of deformations, the calculated curves were significantly lower than the experimental ones, regardless of the indenter diameter. This was caused by the instability of the two-parameter Mooney–Rivlin model applied to the results of the single experiment (one type) when $C_{01}<0$ [38]. This contradicts Drucker’s postulate on the stability of the deformation of inelastic bodies and causes problems in the case of a complex stress state [39]. The illustration of Drucker’s postulate in a formulaic form can be reduced to the following relations [39]:

(14) $$\frac{\partial \sigma_{ij}}{\partial \epsilon_{ij}}\geq 0\;\mathrm{and}\;\partial \sigma_{ij}\partial \epsilon_{ij}\geq 0$$

In particular, for the two-parameter Mooney–Rivlin model, these conditions take the following form [40]:

(15) $$C_{10}+C_{01}\geq 0\;\mathrm{and}\;C_{01}\geq 0$$

**Figure 9:**
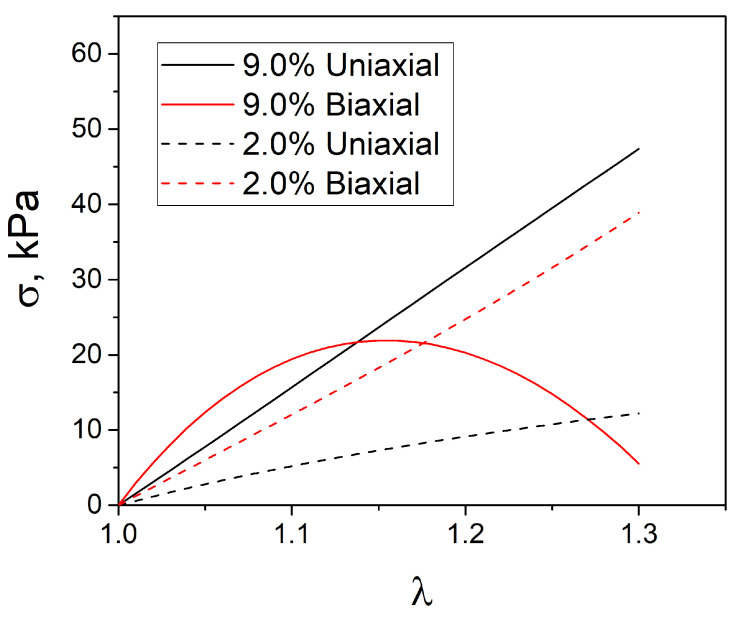
Illustration of the curves for uniaxial and biaxial tension in the Mooney–Rivlin model with constants determined for concentrations of 9% and 2%.

Thus, if $C_{01}<0$, the model is unstable when used in problems where the material is in a complex stress state, which applies to indentation. For the crosslinker concentrations of 2, 1.5, and 1% $C_{01}>0$, the model was therefore stable. The neo-Hookean model was always stable. For additional illustration, we present the curves of uniaxial and biaxial deformation in the Mooney–Rivlin model with constants determined for concentrations of 9% and 2% (Figure 9). It can be seen that, for 9%, starting from a certain strain value, the first derivative of the stress function with respect to strain was less than zero, the incremental work was also less than zero, and, therefore, the model was unstable. For a concentration of 2%, the inequalities resulting from Drucker’s postulate were satisfied over the entire deformation range, so the model was stable.

### 3.8. Biological-Tissue-Mimicking Phantom Prototypes

Based on the results, prototypes for kidney, liver, and spleen phantoms were manufactured. The 3D-printed molds allowed the production of soft biological tissue phantoms with complex geometries by casting. Due to the low viscosity of the gelling precursor solutions, the molds were filled homogeneously.

## 4. Discussion

Hydrogels based on polyacrylamide are prospective materials for soft tissue phantoms. Table 7 shows the tensile elastic modules of the hydrogels evaluated from mechanical tests, and the human soft tissues corresponding with these values. By adjusting the crosslinker concentration, it is possible to obtain materials that reproduce the mechanical properties of biological soft tissues over a wide range.

**Figure 10:**
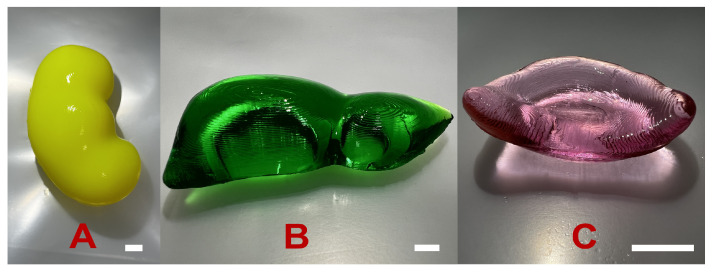
The prototypes for the kidney (**A**), liver (**B**), and spleen (**C**) phantoms. The scale bar is 1 cm.

As an illustration, prototypes of the tissue-mimicking phantoms were obtained by mold casting (see Figure 10). To reproduce the stiffness of the specific biological tissue, the amount of crosslinker was varied according to Table 7.

An important step in the development of a tissue-simulating phantom is the determination of its mechanical properties. The systematic approach proposed in this article not only allows the development of a method for the reproducible production of materials with desirable mechanical properties, but also includes a verified experimental methodology for determining these properties. It opens the possibility of manufacturing and studying not only tissue-simulating phantoms based on polyacrylamide or other polymers, but also various soft materials. Indentation, which is widely used to determine local mechanical properties, has a number of advantages and disadvantages compared to traditional experimental techniques. In particular, indentation does not require the preparation of standardized test specimens or specialized equipment. Modern technology allows mechanical properties to be studied using indenters with diameters down to the nanometer range. At the same time, during indentation, it is necessary to take into account the dimensions of the specimen, including thickness, as well as the influence of edge effects. In the present work, the samples for indentation were prepared in such a way as to minimize the influence of thickness and edge effects on the results of the testing and verification of the elasticity parameters, since the analytical relation for the case of indentation of an elastic half-space was used. If necessary, it is possible to introduce correction functions that take into account the finite thickness of the specimen and its diameter [31,41]. The application of hyperelasticity models, coupled with numerical methods for solving nonlinear problems (both geometrically and physically) allows us to expand the range of problems to be solved and to circumvent the limitations imposed by analytical relations. However, one should keep in mind the peculiarities of the numerical solution. As shown above, the more complex two-parameter Mooney–Rivlin model can give unphysical results when determining constants from a single experiment. The instability of the model can manifest itself depending on the sign of the constant $C_{01}$, which imposes restrictions on this model. This restriction also naturally applies to higher-order models (Ogden, Yeoh), where the physical meaning of the constants is not obvious. Moreover, additional testing is often limited by the thickness of the soft tissue structure, the anisotropy of the mechanical properties, the complexity of setting up and performing the experiment, and the need to use additional equipment. In this case, as shown in the paper, the neo-Hookean one-parameter model gives a good estimate of the mechanical response.

## 5. Conclusions

Although polyacrylamide hydrogels have been well studied, they are still attracting the attention of scientists, not only as a model hydrogel system, but also to find new areas of application. One of the most promising applications is soft-tissue-mimicking phantoms. As with any biomedical application, this requires a detailed understanding of the mechanical properties of the materials. In this work, we investigated the mechanical behavior of polyacrylamide hydrogels with different degrees of crosslinking in tension, torsion, and compression. Using digital image correlation during tensile tests, the Poisson’s ratio was determined to be close to 0.5 for all samples studied. In torsion tests, the magnitude of the initial normal force applied to the specimens, which ranged from 1 to 4 N, was found to have no effect on the shape of the curve within the range of angular strains up to 5%, where the shear modulus G values were determined. The Young’s modulus values obtained in all mechanical tests showed a high correlation. However, for the hydrogels with high crosslinker concentration in the range of 1 to 2%, tension–compression asymmetry was observed, which was not detected for the samples with higher crosslinker density. In addition, the results of the mechanical tests were verified by various indentation tests, including analytical estimation, and full-scale and numerical experiments. We also discussed the limitations of two constitutive models used to fit the uniaxial tensile deformation curves of hydrogels. The neo-Hookean model was stable in the range of the applied deformation for all analyzed degrees of crosslinking, while the two-parameter Mooney–Rivlin model was unstable for the hydrogels with 4.0 and 9.0% crosslinker concentration. The implemented analysis provided a comprehensive approach to the mechanical characterization of biomaterials. The elastic moduli of all investigated hydrogels were in the range of values corresponding to human soft tissues, making them a promising material for phantom application.

## Figures and Tables

**Table 1:** Hydrogel synthesis parameters and degree of swelling.

%C [%]	Crosslinker to Monomer Ratio	α [%]
1.0	1:200	139 ± 8
1.5	1:150	117 ± 3
2.0	1:100	78 ± 9
4.0	1:50	43 ± 6
9.0	1:20	9 ± 2

**Table 2:** Shapes and sizes of the samples.

Mechanical Test	Shape of the Sample	Size, mm
Tension	dumbbell	10 × 20 × 3
Compression	cylinder	d = 16; h = 24
Torsion	cylinder	d = 30; h = 50
Indentation	cylinder	d = 50; h = 30

**Table 3:** Average molecular weight between the crosslinks.

Crosslinking	Swelling	Tension	Theoretical Estimation
%C [%]	$M_{c}$ [kDa]	$M_{c}$ [kDa]	$M_{c}$ [kDa]
1.0	20,788 ± 1448	16,157 ± 1679	14,200
1.5	16,592 ± 433	10,368 ± 998	10,650
2.0	9934 ± 98	6215 ± 782	7100
4.0	5035 ± 770	5108 ± 340	3550
9.0	1822 ± 150	2815 ± 89	1420

**Table 4:** Mechanical properties based on uniaxial tension test.

%C [%]	ν	*E* [kPa]	$\sigma_{B}$ [kPa]	$\epsilon_{B}$ [%]
1.0	0.485	19 ± 2	9 ± 1	66 ± 6
1.5	0.496	31 ± 3	13 ± 1	65 ± 6
2.0	0.477	56 ± 7	22 ± 3	62 ± 6
4.0	0.483	75 ± 5	23 ± 2	33 ± 5
9.0	0.482	157 ± 5	45 ± 5	30 ± 4

**Table 5:** Comparison of the hydrogel elastic parameters obtained in the different tests.

	Tension		Torsion		Compression
%C [%]	ν	E [kPa]	G [kPa]	E [kPa]	E [kPa]
1.0	0.485	19 ± 2	8 ± 1	24 ± 3	30 ± 1
1.5	0.496	31 ± 3	10 ± 1	30 ± 3	42 ± 2
2.0	0.477	56 ± 7	15 ± 1	44 ± 3	69 ± 4
4.0	0.483	75 ± 5	27 ± 1	80 ± 3	86 ± 18
9.0	0.482	157 ± 5	54 ± 3	162 ± 9	163 ± 2

**Table 6:** Shear modules.

	Torsion	Neo-Hook	Mooney–Rivlin		
%C [%]	G [kPa]	G [kPa]	$G=2(C_{10}+C_{01})$ [kPa]	$C_{10}$ [kPa]	$C_{01}$ [kPa]
9.0	54 ± 3	57.5 ± 0.9	51.3 ± 0.3	56.7	−31.0
4.0	27 ± 1	25.4 ± 1.3	24.0 ± 1.0	14.7	−2.4
2.0	15 ± 1	17.2 ± 0.5	20.2 ± 1.3	3.7	6.4
1.5	10 ± 1	10.9 ± 0.5	11.2 ± 0.5	2.9	3.0
1.0	8 ± 1	7.3 ± 0.6	7.5 ± 0.3	1.3	3.0

**Table 7:** Tensile elastic modules of PAAm hydrogels and corresponding soft tissues [21].

%C [%]	*E* [kPa]	Soft Tissue
9.0	157 ± 5	Kidney, uterus
4.0	75 ± 5	Breast, liver
2.0	56 ± 7	Muscle
1.5	31 ± 3	Heart, spleen
1.0	19 ± 2	Tongue, breast

## Data Availability

The raw data supporting the conclusions of this article will be made available by the authors on request.
